# Rising Incidence of Tongue Cancer Surgeries Among Middle‐Aged and Older Women in Japan: A Nationwide Claims‐Based Analysis From 2014 to 2022

**DOI:** 10.1002/cam4.71547

**Published:** 2026-01-18

**Authors:** Masamitsu Kido, Takahiro Tsujikawa, Kengo Yoshii, Shigeyuki Mukudai, Koichi Yoshizawa, Shota Sugaya, Sumiyo Saburi, Katsutoshi Shoda, Alisa Kimura, Shigeru Hirano

**Affiliations:** ^1^ Department of Orthopedic Surgery Inage Hospital Chiba Japan; ^2^ Department of Otolaryngology‐Head and Neck Surgery Kyoto Prefectural University of Medicine Kyoto Japan; ^3^ Department of Mathematics and Statistics in Medical Sciences Kyoto Prefectural University of Medicine Kyoto Japan; ^4^ First Department of Surgery, Faculty of Medicine University of Yamanashi Chuo Japan

**Keywords:** database, epidemiology, Japan, tongue cancer

## Abstract

**Background:**

This study investigated recent trends and demographic patterns in tongue cancer surgeries in Japan using nationwide insurance claims data.

**Methods:**

Annual surgery counts and rates (per 100,000 person‐years) were analyzed using medical (2014–2022) and dental (2016–2022) claims. Trends were evaluated using linear and Poisson regression to estimate annual risk ratios (RRs).

**Results:**

An average of 4470.7 surgeries per year was observed (rate: 3.4). The male‐to‐female ratio was 1.6:1 overall but nearly 1:1 under 40s and over 2.0:1 in the 60s–70s. Surgery rates peaked at ages 75–79 in males (12.0) and 75–84 in females (5.9). Age‐adjusted surgery counts rose significantly from 2014 to 2022. Rates increased significantly among all females (RR = 1.020) and overall (RR = 1.010). Significant increases were seen in females aged 40–44 and 60–64 (RR = 1.083 and 1.055).

**Conclusions:**

Distinct age‐ and sex‐related trends were identified, particularly rising rates among middle‐aged and older females.

## Graphical abstract

This study examines recent epidemiological trends in tongue cancer surgeries in Japan. Using nationwide health insurance claims data from 2014 to 2022, we provide a comprehensive analysis of demographic patterns and surgical rates, revealing a significant rise in tongue cancer surgeries among women. This trend aligns with global observations of increasing tongue cancer incidence among women.

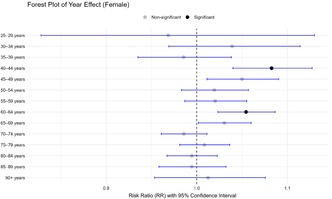

## 1 Introduction

The incidence of tongue cancer has been increasing worldwide [1, 2, 3, 4, 5], with a particularly concerning rise among younger individuals, especially females [2, 3, 6, 7, 8, 9, 10]. According to a 2022 report from the Surveillance, Epidemiology, and End Results (SEER) Program of the U.S. National Cancer Institute, the incidence reached 3.6 per 100,000 person‐years, with an average annual increase of 2.1% between 2013 and 2022 [11]. In Japan, official cancer statistics report the incidence of oral and pharyngeal cancers collectively, rather than tongue cancer specifically [12]. Although the Japan Society for Head and Neck Cancer provides data on new tongue cancer cases, its registry appears limited to participating institutions (e.g., 1382 cases from 159 institutions in 2014 and 2017 cases from 196 institutions in 2021) (Table S1) [13, 14]. Similar to many other countries, the national incidence of tongue cancer in Japan is presumably increasing.

Japan's universal healthcare system enables near‐complete coverage of medical services, allowing for large‐scale epidemiological research. The National Database of Health Insurance Claims and Specific Health Checkups of Japan (NDB) [15], which includes more than 95% of all insurance claims, is a comprehensive and highly representative source of real‐world data. Recent studies have suggested that analyses based on procedural codes rather than diagnostic codes may improve the accuracy of database‐driven research [16]. Our previous study demonstrated the feasibility of procedure‐based epidemiological analysis for conditions with sampling challenges [17, 18, 19, 20].

Glossectomy is the standard surgical treatment for tongue cancer across all stages [21], suggesting that the number of tongue cancer surgeries may serve as a practical proxy for estimating new tongue cancer cases. In this study, we aimed to address the gap in tongue cancer epidemiological data in Japan by analyzing nationwide trends in tongue cancer surgeries using the NDB. This approach may help overcome limitations associated with traditional cancer registries. Furthermore, we sought to clarify sex‐ and age‐specific demographic patterns associated with tongue cancer surgery to enhance our understanding of the epidemiological profile of tongue cancer in Japan.

## 2 Methods

### 2.1 Study Design and Data Sources

In accordance with Japanese ethical guidelines, Institutional Review Board approval and informed consent were not required because all data were obtained from publicly available sources. To protect patient anonymity, the publicly available NDB Open Data Japan [22] does not report procedural data when the annual case count is less than 10.

Data on tongue cancer surgeries, stratified by sex and 5‐year age groups, were extracted annually from 2014 to 2022 using the NDB Open Data Japan database. Both inpatient and outpatient procedures were included in the study. In Japan, tongue cancer surgeries are performed by otolaryngologists or oral surgeons and are recorded under either medical or dental procedure codes. Medical claims data were available for the full period (2014–2022), whereas dental claims data were available from 2016 to 2022. Demographic data from the Ministry of Internal Affairs and Communications [23] were used to calculate the procedure rates per 100,000 person‐years.

### 2.2 Procedure Codes Used to Identify Tongue Cancer Surgeries

The following procedure codes were used to identify tongue cancer surgeries:

K415 Malignant tongue tumor surgery (medical claims)

1. Partial glossectomy: Resection of a portion of the mobile tongue, typically for localized malignant tumors.
2. Subtotal or greater glossectomy: Extensive resection of tongue tumors requiring removal of a substantial portion of the tongue.

J018 Malignant tongue tumor surgery (dental claims)

1. Partial glossectomy: Same as above.
2. Subtotal or greater glossectomy: Same as above.

### 2.3 Data Analyses

Using the available data on both medical and dental tongue cancer surgeries from 2016 to 2022, overall and age‐stratified male‐to‐female (M/F) ratios were calculated. A demographic peak analysis was conducted on sex‐ and age‐stratified procedure rates per 100,000 person‐years.

Annual trends were evaluated based on the number and rate of each procedure. Annual trends in the number of procedures were analyzed using linear regression models applied to medical datasets from 2014 to 2022 and to dental datasets from 2016 to 2022. In addition, annual trends in the rate of each procedure were evaluated using Poisson regression models applied to medical data only, due to the limited time span of dental records. Risk ratios (RRs) were calculated for the overall cohorts and age‐stratified subgroups by sex, with RRs > 1.0 indicating a yearly increase and RRs < 1.0 indicating a yearly decrease in procedure rates per 100,000 population.

### 2.4 Statistical Analyses

Age was adjusted using a direct method based on the 2015 standard Japanese population [19]. The linear regression models were used to determine whether the number of procedures increased or decreased over the study period.

Poisson regression models were constructed to evaluate the annual trends in age‐adjusted procedure rates per 100,000 person‐years. The number of each procedure was set as the objective variable, and the observation time‐point (years) and sex were set as explanatory variables. Additionally, the population at each time point was considered by adding the person‐year population to the model as an offset. The number was also adjusted for age using a direct method with the same age structure [19]. Moreover, an annual trend analysis of the subgroups was performed using age‐ and sex‐stratified samples.

All statistical analyses were performed using R version 3.6.2 Statistical Computing Program (R Foundation; www.r‐project.org). Statistical significance was set at two‐sided *p*‐values of < 0.05 for linear regression analyses, and < 0.0167 (0.05/3) and < 0.00088 (0.05/57) for the Poisson regression models for all‐ages and age‐stratified analyses, respectively, using the Bonferroni correction method for multiple comparisons.

## 3 Results

### 3.1 Comprehensive Analysis of Tongue Cancer Surgeries

Based on medical and dental datasets from 2016 to 2022, the mean annual number of tongue cancer surgeries was 4470.7 procedures, with a rate of 3.4 per 100,000 person‐years (Table S2). The overall male‐to‐female (M/F) ratio was 1.6:1, indicating a moderate male predominance.

**FIGURE 1:**
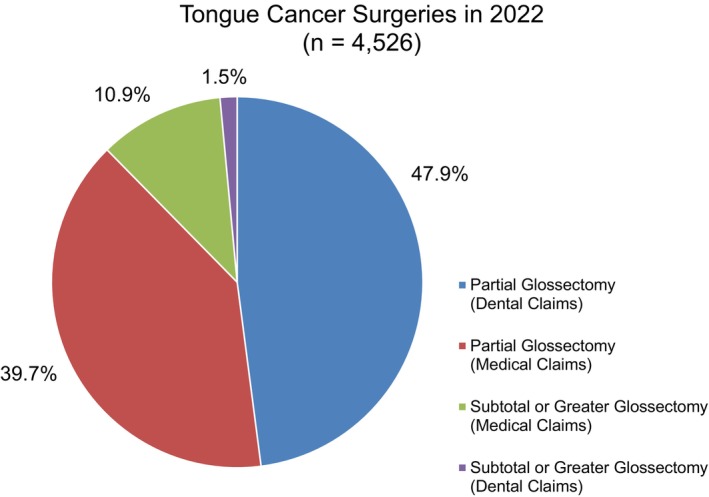
Annual mode share of tongue cancer surgeries in 2022. In 2022, 4526 tongue cancer surgeries were identified: 2170 partial glossectomies (dental claims, 47.9%), 1797 partial glossectomies (medical claims, 39.7%), 492 subtotal or greater glossectomies (medical claims, 10.9%), and 67 subtotal or greater glossectomies (dental claims, 1.5%).

In 2022, 4526 tongue cancer surgeries were identified: 2170 partial glossectomies (dental claims, 47.9%), 1797 partial glossectomies (medical claims, 39.7%), 492 subtotal or greater glossectomies (medical claims, 10.9%), and 67 subtotal or greater glossectomies (dental claims, 1.5%) (Figure 1).

### 3.2 Demographic Peak Analysis of Tongue Cancer Surgeries (2016–2022)

**FIGURE 2:**
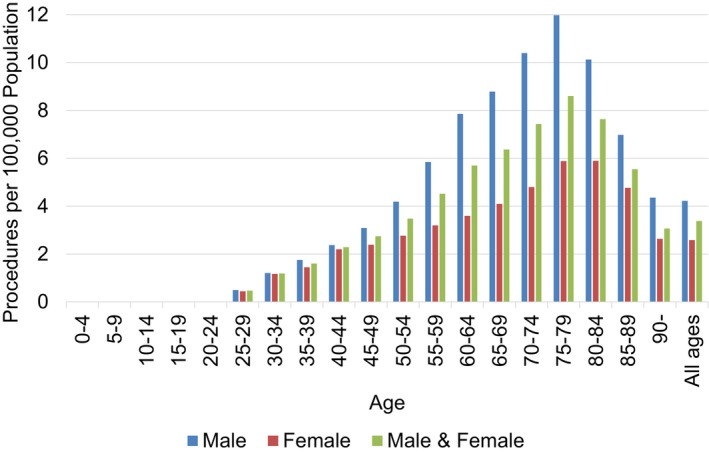
Age‐stratified number rate of tongue cancer surgeries per 100,000 population in Japan (males, females, and both sexes, 2016–2022).

Throughout the study period, the age‐stratified procedures exhibited a unimodal peak pattern in males and females (Figure 2). In males, the peak was observed at ages 75–79 years (12.0 procedures per 100,000 person‐years), whereas in females, the peak was noted at ages 75–84 years (5.9 procedures per 100,000 person‐years). Eventually, in both sexes, a peak was observed at ages 75–79 years (8.6 procedures per 100,000 person‐years). In each age group, male dominance in the procedure numbers was observed compared to females.

**FIGURE 3:**
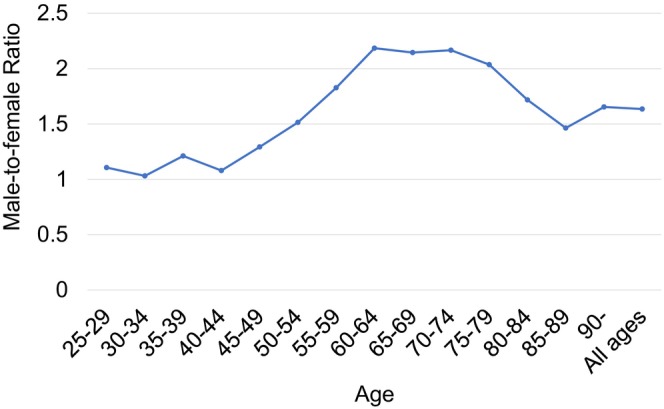
Age‐stratified male‐to‐female ratio of tongue cancer surgeries per 100,000 population (2016–2022).

The age‐stratified M/F ratio was approximately 1.0:1 among individuals under 40s (Figure 3). From 45 to 49 years onward, the ratio gradually increased with age, peaking above 2.0:1 in the 60s–70s age group, indicating a marked male predominance. After the age of 80 years, the ratio declined slightly to 1.5–1.7:1, though males continued to outnumber females.

### 3.3 Trends in the Annual Number of Tongue Cancer Surgeries

**FIGURE 4:**
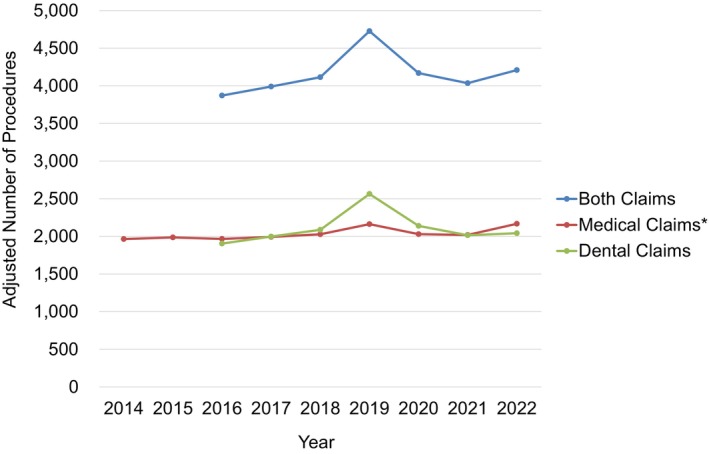
Trends in the annual age‐adjusted number of tongue cancer surgeries in Japan from 2014 to 2022. * indicates a statistically significant trend (*p* < 0.05).

A significant upward trend was observed in the age‐adjusted number of medical tongue cancer surgeries performed between 2014 and 2022 (*p* < 0.05) (Figure 4). In contrast, no significant trends were observed in the number of dental tongue cancer surgeries or in the combined total number of medical and dental procedures between 2016 and 2022 (*p* = 0.701 and *p* = 0.477, respectively).

### 3.4 Trends in the Age‐Adjusted Rate of Tongue Cancer Surgeries (2014–2022)

**FIGURE 5:**
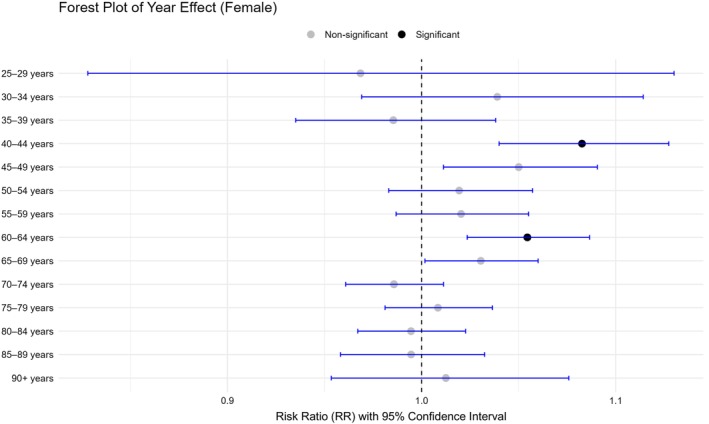
Risk ratio of the age‐stratified number of tongue cancer surgeries among females per 100,000 person‐years using fitting Poisson regression models. CI, confidence interval; RR, risk ratio. Black dots represent significant, and gray dots represent non‐significant.

The age‐adjusted rates across all age groups per 100,000 person‐years demonstrated a statistically significant upward trend in females (RR = 1.020; *p* < 0.0167) and in both sexes combined (RR = 1.010; *p* < 0.0167) (Table 1). Subgroup analyses showed significant annual increases in the 40–44 and 60–64 year age groups among females (RR = 1.083 and 1.055, respectively; *p* < 0.00088) (Figure 5 and Table 2).

**TABLE 1:** Annual trend analysis of the age‐adjusted number of tongue cancer surgeries across all ages per 100,000 person‐years using fitting Poisson regression models. A (upper): Poisson regression model for males; B (middle): Poisson regression model for females; C (lower): Poisson regression model for both sexes, considering the effect of sex.

A				
Variables	RR	95% CI (low)	95% CI (high)	*p*
Constant	0.029	0.028	0.030	< 0.0001
Time‐point	1.004	0.998	1.011	0.1960

B				
Variables	RR	95% CI (low)	95% CI (high)	*p*
Constant	0.013	0.012	0.013	< 0.0001
Time‐point	1.020	1.010	1.030	< 0.0001

C				
Variables	RR	95% CI (low)	95% CI (high)	*p*
Constant	0.020	0.020	0.020	< 0.0001
Time‐point	1.010	1.004	1.016	0.0006

Abbreviations: CI, confidence interval; RR, risk ratio.

**TABLE 2:** Annual trend analysis of the age‐stratified number of tongue cancer surgeries of males, females, and both sexes per 100,000 person‐years by fitting Poisson regression models.

Sex	Age group	Variables	RR	95% CI (low)	95% CI (high)	*p*
Male	25–29 years	Constant	0.001	0.001	0.002	< 0.0001
Male	25–29 years	Time point	1.026	0.919	1.146	0.6535
Male	30–34 years	Constant	0.006	0.005	0.008	< 0.0001
Male	30–34 years	Time point	0.980	0.926	1.037	0.4875
Male	35–39 years	Constant	0.008	0.007	0.010	< 0.0001
Male	35–39 years	Time point	0.988	0.945	1.033	0.5983
Male	40–44 years	Constant	0.011	0.009	0.013	< 0.0001
Male	40–44 years	Time point	1.037	1.003	1.073	0.0343
Male	45–49 years	Constant	0.015	0.013	0.018	< 0.0001
Male	45–49 years	Time point	1.021	0.992	1.051	0.1570
Male	50–54 years	Constant	0.020	0.017	0.023	< 0.0001
Male	50–54 years	Time point	1.023	0.997	1.051	0.0831
Male	55–59 years	Constant	0.035	0.031	0.038	< 0.0001
Male	55–59 years	Time point	0.990	0.968	1.012	0.3782
Male	60–64 years	Constant	0.039	0.036	0.043	< 0.0001
Male	60–64 years	Time point	1.019	0.999	1.039	0.0627
Male	65–69 years	Constant	0.050	0.046	0.054	< 0.0001
Male	65–69 years	Time point	0.988	0.970	1.006	0.1913
Male	70–74 years	Constant	0.059	0.054	0.064	< 0.0001
Male	70–74 years	Time point	0.994	0.977	1.011	0.4633
Male	75–79 years	Constant	0.061	0.056	0.067	< 0.0001
Male	75–79 years	Time point	1.000	0.982	1.019	0.9615
Male	80–84 years	Constant	0.045	0.04	0.051	< 0.0001
Male	80–84 years	Time point	1.028	1.003	1.053	0.0264
Male	85–89 years	Constant	0.025	0.020	0.031	< 0.0001
Male	85–89 years	Time point	1.018	0.975	1.064	0.4196
Male	90+ years	Constant	0.023	0.016	0.033	< 0.0001
Male	90+ years	Time point	0.981	0.909	1.059	0.6239
Female	25–29 years	Constant	0.001	0.000	0.002	< 0.0001
Female	25–29 years	Time point	0.969	0.828	1.130	0.6846
Female	30–34 years	Constant	0.003	0.002	0.005	< 0.0001
Female	30–34 years	Time point	1.039	0.969	1.114	0.2814
Female	35–39 years	Constant	0.006	0.005	0.008	< 0.0001
Female	35–39 years	Time point	0.985	0.935	1.038	0.5820
Female	40–44 years	Constant	0.007	0.005	0.008	< 0.0001
Female	40–44 years	Time point	1.083	1.040	1.127	0.0001
Female	45–49 years	Constant	0.008	0.007	0.010	< 0.0001
Female	45–49 years	Time point	1.050	1.011	1.091	0.0111
Female	50–54 years	Constant	0.011	0.009	0.013	< 0.0001
Female	50–54 years	Time point	1.019	0.983	1.057	0.2995
Female	55–59 years	Constant	0.014	0.011	0.016	< 0.0001
Female	55–59 years	Time point	1.020	0.987	1.055	0.2370
Female	60–64 years	Constant	0.014	0.012	0.016	< 0.0001
Female	60–64 years	Time point	1.055	1.024	1.087	0.0005
Female	65–69 years	Constant	0.016	0.014	0.018	< 0.0001
Female	65–69 years	Time point	1.031	1.002	1.060	0.0372
Female	70–74 years	Constant	0.023	0.020	0.026	< 0.0001
Female	70–74 years	Time point	0.986	0.961	1.011	0.2717
Female	75–79 years	Constant	0.022	0.020	0.026	< 0.0001
Female	75–79 years	Time point	1.008	0.981	1.036	0.5476
Female	80–84 years	Constant	0.027	0.023	0.031	< 0.0001
Female	80–84 years	Time point	0.995	0.967	1.023	0.7004
Female	85–89 years	Constant	0.021	0.017	0.025	< 0.0001
Female	85–89 years	Time point	0.995	0.958	1.032	0.7761
Female	90+ years	Constant	0.010	0.008	0.014	< 0.0001
Female	90+ years	Time point	1.013	0.954	1.076	0.6852
Male and Female	25–29 years	Constant	0.001	0.001	0.002	< 0.0001
Male and Female	25–29 years	Time point	1.006	0.920	1.101	0.8926
Male and Female	30–34 years	Constant	0.005	0.004	0.006	< 0.0001
Male and Female	30–34 years	Time point	1.003	0.960	1.048	0.8816
Male and Female	35–39 years	Constant	0.007	0.006	0.009	< 0.0001
Male and Female	35–39 years	Time point	0.987	0.954	1.021	0.4502
Male and Female	40–44 years	Constant	0.009	0.008	0.010	< 0.0001
Male and Female	40–44 years	Time point	1.056	1.029	1.083	< 0.0001
Male and Female	45–49 years	Constant	0.012	0.010	0.013	< 0.0001
Male and Female	45–49 years	Time point	1.032	1.009	1.056	0.0072
Male and Female	50–54 years	Constant	0.015	0.014	0.017	< 0.0001
Male and Female	50–54 years	Time point	1.022	1.001	1.044	0.0424
Male and Female	55–59 years	Constant	0.024	0.022	0.026	< 0.0001
Male and Female	55–59 years	Time point	1.000	0.981	1.018	0.9624
Male and Female	60–64 years	Constant	0.026	0.024	0.029	< 0.0001
Male and Female	60–64 years	Time point	1.030	1.013	1.047	0.0004
Male and Female	65–69 years	Constant	0.032	0.030	0.035	< 0.0001
Male and Female	65–69 years	Time point	1.000	0.985	1.016	0.9508
Male and Female	70–74 years	Constant	0.039	0.037	0.042	< 0.0001
Male and Female	70–74 years	Time point	0.992	0.979	1.006	0.2778
Male and Female	75–79 years	Constant	0.04	0.037	0.043	< 0.0001
Male and Female	75–79 years	Time point	1.004	0.988	1.019	0.6542
Male and Female	80–84 years	Constant	0.034	0.031	0.037	< 0.0001
Male and Female	80–84 years	Time point	1.015	0.996	1.034	0.1135
Male and Female	85–89 years	Constant	0.022	0.019	0.026	< 0.0001
Male and Female	85–89 years	Time point	1.005	0.977	1.035	0.7074
Male and Female	90+ years	Constant	0.013	0.010	0.017	< 0.0001
Male and Female	90+ years	Time point	1.004	0.957	1.052	0.8800

Abbreviations: CI, confidence interval; RR, risk ratio.

## 4 Discussion

This nationwide observational epidemiological study analyzed tongue cancer surgeries according to sex and age, using comprehensive health insurance claims data from Japan. Our findings revealed distinct sex‐ and age‐related patterns and a gradual increase in tongue cancer surgeries over the past decade. The overall age‐adjusted tongue cancer surgery rate was 3.4 per 100,000 person‐years, comparable to tongue cancer incidence rates reported in Western countries, such as the United States (3.6) [11] and Australia (4.4) [5]. The overall male‐to‐female (M/F) ratio of tongue cancer surgeries was 1.6:1, closely aligning with the SEER‐reported incidence ratio for non‐Hispanic Asian/Pacific Islanders (1.8:1), but lower than the overall U.S. ratio across all racial groups (2.6:1) [11].

Demographic analysis revealed a unimodal distribution of tongue cancer surgeries, with peaks in the 70s–80s age group for males and females (Figure 2), consistent with global trends showing a higher tongue cancer incidence among older adults [1, 4, 11]. Age‐stratified M/F ratios showed near parity in patients under 40s (1.0:1), increasing to over 2.0:1 in individuals in their 60s–70s and slightly declining after the age of 80 years (to 1.5–1.7:1). This pattern may reflect cumulative exposure to traditional risk factors such as smoking and alcohol use, which are more prevalent in older male cohorts. Previous studies have reported a wide range of M/F ratios (0.9–14:1) for tongue cancer incidence, often based on small or biased samples [9, 24, 25]. In contrast, our study utilized large‐scale, nationally representative data, enabling a more precise and age‐stratified analysis of sexual disparities. Notably, a supplementary analysis using publicly available, but limited, data on the tongue cancer incidence in Japan from 2014 to 2021 [13] produced comparable findings (Figures S1 and S2), supporting the robustness of our results.

Increasing trends in the number and rate of medical tongue cancer surgeries are consistent with global observations of an increasing tongue cancer incidence [1, 2, 3, 4, 5]. Notably, in the detailed analysis, we observed significant increases among females aged 40–44 and 60–64 years, reflecting broader concerns about rising tongue cancer rates among younger and older women [2, 3, 6, 7, 8, 9, 10]. Recent U.S. SEER data also suggest a rising incidence among individuals aged over 50 years, particularly among non‐Hispanic Whites, indicating potential shifts in tongue cancer epidemiology [26]. Notably, tongue cancers in women are reported to exhibit distinct genetic alterations, such as *TERT* promoter and *MYH9* mutations, suggesting that female tongue cancers are clinically and biologically distinct [27]. The trends identified in our study suggest that women in these age groups may represent emerging high‐risk cohorts that warrant close public health attention.

These findings highlight the need for age‐ and sex‐specific public health interventions. The observed increase in middle‐aged and older female cases highlights the importance of targeted prevention strategies, including public education on modifiable risk factors, such as tobacco and alcohol use. The persistent male predominance in the older age groups supports the integration of tongue cancer screening into routine health checkups for high‐risk populations. Furthermore, the unimodal age peak in the older adult population suggests that geriatric‐oriented surgical and rehabilitative care should be prioritized in clinical management.

This study had several limitations. First, because this analysis was based on health insurance claims data, potential sources of bias such as population selection bias, information bias, coding inaccuracies, and disease misclassification cannot be completely excluded. Although the NDB covers more than 95% of all medical and dental claims in Japan, and thus the magnitude of selection bias is expected to be small, the data are primarily collected for administrative and reimbursement purposes rather than for clinical research. Second, tongue cancer surgery counts may overestimate the true incidence due to potential reoperations, procedures for non‐squamous malignancies (e.g., lymphoma and sarcoma), and excision of potentially malignant oral disorders. Third, data on patient background, such as cancer stage, comorbidities, and behavioral risk factors (e.g., smoking and alcohol use), were not available. Fourth, the shorter observation period for dental claims (2016–2022) limited the scope of the trend analysis across the entire dataset. Finally, the generalizability to other countries may be limited owing to differences in healthcare systems, population demographics, and cultural factors.

## 5 Conclusions

The average annual number of tongue cancer surgeries was 3.4 per 100,000 person‐years. The male‐to‐female ratio was 1.6:1 overall but nearly 1:1 under 40s and over 2.0:1 in the 60s–70s. Age‐adjusted procedure rates increased over time in the overall cohort, particularly among females, with notable increases in the 40–44 and 60–64 year age groups. These findings provide valuable insights into the epidemiology of tongue cancer surgeries in Japan and may enhance the broader understanding of tongue cancer profiles.

## Author Contributions

**Masamitsu Kido:** conceptualization (equal), data curation (equal), formal analysis (equal), investigation (equal), methodology (equal), visualization (equal), writing – original draft (equal). **Takahiro Tsujikawa:** conceptualization (equal), formal analysis (equal), funding acquisition (equal), investigation (equal), methodology (equal), project administration (equal), visualization (equal), writing – review and editing (equal). **Kengo Yoshii:** formal analysis (equal), methodology (equal), validation (equal), visualization (equal), writing – review and editing (equal). **Shigeyuki Mukudai:** investigation (equal), writing – review and editing (equal). **Koichi Yoshizawa:** investigation (equal), writing – review and editing (equal). **Katsutoshi Shoda:** methodology (equal), writing – review and editing (equal). **Alisa Kimura:** writing – review and editing (equal). **Shigeru Hirano:** supervision (equal). **Shota Sugaya:** writing – review and editing (equal). **Sumiyo Saburi:** funding acquisition (equal), writing – review and editing (equal).

## Funding

This work was supported by  the Japan Society for the Promotion of Science (22K09688, and 25K20167).

## Ethics Statement

This study utilized publicly available databases.

## Conflicts of Interest

The authors declare no conflicts of interest.

## Supporting information


**Figure S1:** Age‐stratified incidence rate of carcinoma of the tongue per 100,000 population in Japan (2014–2021). This figure was created based on publicly available data [13, 23].
**Figure S2:** Age‐stratified male‐to‐female ratio of incidence rate of carcinoma of the tongue per 100,000 population in Japan (2014–2021). This figure was created based on publicly available data [13, 23].

**Table S1:** Data on new cases of tongue carcinoma (Japan Society for Head and Neck Cancer).
**Table S2:** Annual counts of tongue cancer surgeries by claims type (2016–2022).

## Data Availability

The data that support the findings of this study are available in Open Data NDB at https://www.mhlw.go.jp/stf/seisakunitsuite/bunya/0000177182.html. These data were derived from the following resources available in the public domain: Open Data NDB, https://www.mhlw.go.jp/stf/seisakunitsuite/bunya/0000177182.html—Portal site of official statistics of Japan (e‐stat), https://www.e‐stat.go.jp/en/stat‐search/—Report of National Malignant Tumor Registries, http://www.jshnc.umin.ne.jp/report.html—Surveillance, Epidemiology, and End Results (SEER) Program, https://seer.cancer.gov/statfacts/html/tongue.html—Gan Joho Service, https://ganjoho.jp/reg_stat/statistics/stat/cancer/3_oral.html—Use of National Database of Health Insurance Claims and Specific Health Checkups of Japan (NDB), https://www.mhlw.go.jp/stf/seisakunitsuite/bunya/kenkou_iryou/iryouhoken/reseputo/index.html.
