# Supplementary material for: Rising Incidence of Tongue Cancer Surgeries Among Middle‐Aged and Older Women in Japan: A Nationwide Claims‐Based Analysis From 2014 to 2022
Source: Cancer Med. 2026 Jan 18;15(1):e71547. doi: 10.1002/cam4.71547 (PMC12812491; doi:10.1002/cam4.71547)
Supplement: Supplementary file 1 — Figure S1: Age‐stratified incidence rate of carcinoma of the tongue per 100,000 population in Japan (2014–2021). This figure was created based on publicly available data [13, 23]. Figure S2: Age‐stratified male‐to‐female ratio of incidence rate of carcinoma of the tongue per 100,000 population in Japan (2014–2021). This figure was created based on publicly available data [13, 23]. [file CAM4-15-e71547-s001.docx]

Supplementary Figure 1. Age-stratified incidence rate of carcinoma of the tongue per 100,000 population in Japan (2014-2021).

This figure was created based on publicly available data [13, 23].

Supplementary Figure 2. Age-stratified male-to-female ratio of incidence rate of carcinoma of the tongue per 100,000 population in Japan (2014-2021).

This figure was created based on publicly available data [13, 23].
