# Supplementary material for: Rising Incidence of Tongue Cancer Surgeries Among Middle‐Aged and Older Women in Japan: A Nationwide Claims‐Based Analysis From 2014 to 2022
Source: Cancer Med. 2026 Jan 18;15(1):e71547. doi: 10.1002/cam4.71547 (PMC12812491; doi:10.1002/cam4.71547)
Supplement: Supplementary file 2 — Table S1: Data on new cases of tongue carcinoma (Japan Society for Head and Neck Cancer). Table S2: Annual counts of tongue cancer surgeries by claims type (2016–2022). [file CAM4-15-e71547-s002.docx]

**Supplementary materials**

**Supplementary Table 1. Data on new cases of tongue carcinoma (Japan Society for Head and Neck Cancer)**

| Year | Participating institutions | Sex | Age group |  |  |  |  |  |  |  |  |  | Overall |
| --- | --- | --- | --- | --- | --- | --- | --- | --- | --- | --- | --- | --- | --- |
|  |  |  | <10 | 10s | 20s | 30s | 40s | 50s | 60s | 70s | 80s | 90< |  |
| 2014 | 159 | Male | 0 | 0 | 15 | 58 | 89 | 170 | 257 | 239 | 83 | 8 | 919 |
|  |  | Female | 0 | 1 | 12 | 38 | 43 | 70 | 109 | 120 | 58 | 12 | 463 |
|  |  | Total | 0 | 1 | 27 | 96 | 132 | 240 | 366 | 359 | 141 | 20 | 1382 |
| 2015 | 173 | Male | 0 | 2 | 13 | 47 | 86 | 143 | 235 | 227 | 61 | 8 | 822 |
|  |  | Female | 0 | 0 | 7 | 37 | 57 | 71 | 92 | 94 | 69 | 3 | 430 |
|  |  | Total | 0 | 2 | 20 | 84 | 143 | 214 | 327 | 321 | 130 | 11 | 1252 |
| 2016 | 184 | Male | 0 | 2 | 23 | 46 | 122 | 174 | 314 | 283 | 92 | 4 | 1060 |
|  |  | Female | 0 | 1 | 17 | 41 | 68 | 85 | 122 | 113 | 99 | 13 | 559 |
|  |  | Total | 0 | 3 | 40 | 87 | 190 | 259 | 436 | 396 | 191 | 17 | 1619 |
| 2017 | 193 | Male | 2 | 2 | 22 | 64 | 126 | 183 | 362 | 277 | 113 | 5 | 1156 |
|  |  | Female | 1 | 2 | 17 | 44 | 77 | 97 | 136 | 166 | 96 | 9 | 645 |
|  |  | Total | 3 | 4 | 39 | 108 | 203 | 280 | 498 | 443 | 209 | 14 | 1801 |
| 2018 | 201 | Male | 3 | 1 | 25 | 63 | 132 | 202 | 355 | 362 | 153 | 10 | 1306 |
|  |  | Female | 0 | 2 | 13 | 36 | 93 | 106 | 133 | 165 | 115 | 13 | 676 |
|  |  | Total | 3 | 3 | 38 | 99 | 225 | 308 | 488 | 527 | 268 | 23 | 1982 |
| 2019 | 205 | Male | 1 | 1 | 24 | 70 | 165 | 234 | 343 | 365 | 124 | 10 | 1337 |
|  |  | Female | 0 | 3 | 23 | 51 | 122 | 131 | 146 | 197 | 127 | 27 | 827 |
|  |  | Total | 1 | 4 | 47 | 121 | 287 | 365 | 489 | 562 | 251 | 37 | 2164 |
| 2020 | 215 | Male | 1 | 3 | 24 | 51 | 143 | 171 | 279 | 345 | 136 | 14 | 1167 |
|  |  | Female | 0 | 2 | 16 | 41 | 100 | 127 | 124 | 169 | 106 | 21 | 706 |
|  |  | Total | 1 | 5 | 40 | 92 | 243 | 298 | 403 | 514 | 242 | 35 | 1873 |
| 2021 | 196 | Male | 1 | 4 | 28 | 72 | 132 | 235 | 316 | 370 | 138 | 10 | 1306 |
|  |  | Female | 1 | 0 | 20 | 57 | 97 | 119 | 118 | 165 | 114 | 20 | 711 |
|  |  | Total | 2 | 4 | 48 | 129 | 229 | 354 | 434 | 535 | 252 | 30 | 2017 |

This table was created based on publicly available data [13].

In Japan, a hospital is defined as a facility that can accommodate ≥20 patients, and a general hospital is defined as a hospital excluding psychiatric hospitals and tuberculosis sanatoriums. According to the administrative nationwide survey (https://www.e-stat.go.jp/dbview?sid=0004003787) [23], there were 7,426 general hospitals in Japan in 2014, and 7,152 in 2021.

**Supplementary Table 2. Annual counts of tongue cancer surgeries by claims type (2016-2022)**

| Year | Total Surgeries | Partial Glossectomy | | Subtotal/Greater Glossectomy | |
| --- | --- | --- | --- | --- | --- |
|  |  | Medical Claims | Dental Claims | Medical Claims | Dental Claims |
| 2016 | 4,090 | 1,556 | 1,907 | 519 | 108 |
| 2017 | 4,245 | 1,615 | 2,054 | 497 | 79 |
| 2018 | 4,444 | 1,710 | 2,165 | 481 | 88 |
| 2019 | 5,078 | 1,840 | 2,676 | 489 | 73 |
| 2020 | 4,515 | 1,667 | 2,272 | 495 | 81 |
| 2021 | 4,397 | 1,667 | 2,166 | 503 | 61 |
| 2022 | 4,526 | 1,797 | 2,170 | 492 | 67 |
| Mean | 4,470.7 | 1,680.3 | 2,192.9 | 495.4 | 79.6 |
